# 2-*exo*,5-*endo*,8,8,10-Penta­chloro­bornane

**DOI:** 10.1107/S1600536808006235

**Published:** 2008-03-12

**Authors:** Arto Valkonen, Erkki Kolehmainen, Vladimir Nikiforov

**Affiliations:** a University of Jyväskylä, Department of Chemistry, PO Box 35, FIN-40014 Jyväskylä, Finland; b St Petersburg University, Department of Chemistry, 198504, Petrodvorets, Universitetskii pr. 26, St Petersburg, Russian Federation

## Abstract

The title compound, C_10_H_13_Cl_5_, is a polychlorinated monoterpene and a Toxaphene congener. This compound is also the only penta­chlorinated derivative of camphene formed *via* ionic chlorination. Previously, the title compound was thought to be 2-*exo*,5-*endo*,9,9,10-penta­chloro­bornane, but X-ray structural analysis showed it to have a different structure and rather to be 2-*exo*,5-*endo*,8,8,10-penta­chloro­bornane. The title compound shows static disorder and almost half the molecule was divided in two partitions with an occupancy ratio of 0.575 (major) to 0.425 (minor). The repulsive close contacts of Cl atoms could possibly be the cause for this disorder.

## Related literature

For the preparation of 6-*exo*-chloro­camphene and further the title compound, see: Jennings & Herschbach (1965 ▶). For the background and related compounds, see: Nikiforov *et al.* (1999 ▶, 2000 ▶, 2001 ▶).
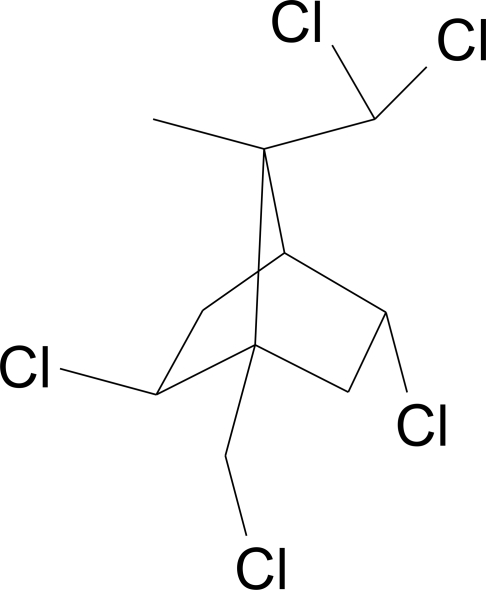


## Experimental

### 

#### Crystal data

C_10_H_13_Cl_5_
*M* *_r_* = 310.45
Orthorhombic, 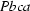

*a* = 12.2386 (2) Å
*b* = 9.07010 (10) Å
*c* = 23.0822 (3) Å
*V* = 2562.25 (6) Å^3^
*Z* = 8
Mo *K*α radiation
μ = 1.10 mm^−1^
*T* = 173 (2) K
0.24 × 0.16 × 0.10 mm

#### Data collection

Bruker Kappa APEXII diffractometer
Absorption correction: multi-scan (*MULABS* in *PLATON*; Blessing, 1995 ▶; Spek, 2003 ▶) *T*_min_ = 0.779, *T*_max_ = 0.898
35431 measured reflections
2612 independent reflections
2440 reflections with *I* > 2σ(*I*)
*R*_int_ = 0.064

#### Refinement

*R*[*F* ^2^ > 2σ(*F* ^2^)] = 0.091
*wR*(*F* ^2^) = 0.191
*S* = 1.25
2612 reflections
185 parameters
167 restraints
H-atom parameters constrained
Δρ_max_ = 0.88 e Å^−3^
Δρ_min_ = −0.80 e Å^−3^

### 

Data collection: *COLLECT* (Bruker, 2004 ▶); cell refinement: *DENZO-SMN* (Otwinowski & Minor, 1997 ▶) and *DIRAX* (Duisenberg,1992 ▶); data reduction: *DENZO-SMN*; program(s) used to solve structure: *SIR2002* (Burla *et al.*, 2003 ▶); program(s) used to refine structure: *SHELXL97* (Sheldrick, 2008 ▶); molecular graphics: *ORTEP-3 for Windows* (Farrugia, 1997 ▶); software used to prepare material for publication: *SHELXL97* (Sheldrick, 2008 ▶) and *Mercury* (Macrae *et al.*, 2006 ▶).

## Supplementary Material

Crystal structure: contains datablocks global, I. DOI: 10.1107/S1600536808006235/zl2100sup1.cif

Structure factors: contains datablocks I. DOI: 10.1107/S1600536808006235/zl2100Isup2.hkl

Additional supplementary materials:  crystallographic information; 3D view; checkCIF report

## Figures and Tables

**Fig. 1.**
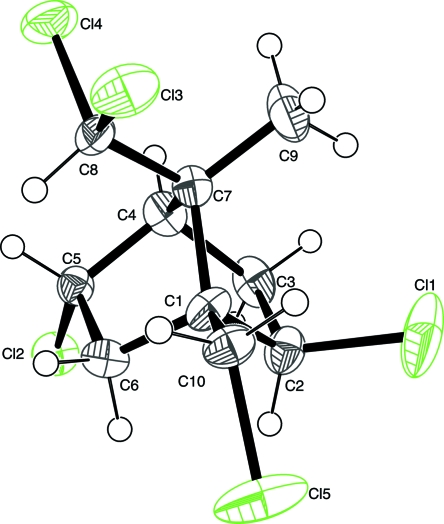
View of the molecule of title compound showing the atom-labeling scheme. Displacement ellipsoids are drawn at the 50% probability level. H atoms are represented by circles of arbitrary size. The disorder was removed from the figure, showing only the major component.

**Fig. 2.**
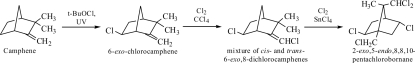
The preparation of title compound.

**Fig. 3.**
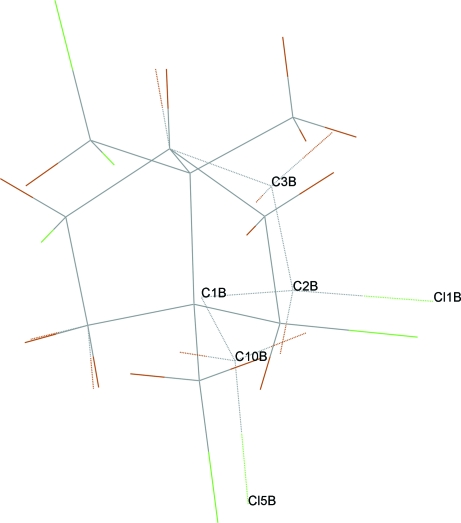
Wireframe view of the title compound, showing the major and minor components.

## supplementary crystallographic information

### Comment

The title compound (Fig 1) of this study is a polychlorinated monoterpene and a Toxaphene congener. It is prepared *via* ionic chlorination of 6-*exo*-chlorocamphene in solution in carbon tetrachloride, followed by chlorination of intermediate dichlorocamphene in presence of Lewis acid (Fig 2). The last transformation obviously involves a series of carbocationic rearrangements, but details of the exact mechanism remain unknown. Based on analytical and mechanistic data the compound was previously proposed to be 2-*exo*,5-*endo*,9,9,10-pentachlorobornane (Nikiforov *et al.*, 2001; Nikiforov *et al.*, 2000; Nikiforov *et al.*, 1999), but this was not verified by crystallographic data and so we decided to perform a detailed single-crystal difffraction analysis to unambiguously identify the nature of the pentachlorinated compound. The structure obtained, however, was found to be different from the previously suggested one. The dichloromethyl and methyl groups at carbon atom C7 exhibit a different orientation than previously presumed for 2-*exo*,5-*endo*,9,9,10-pentachlorobornane, and the correct name of the title compound should thus be 2-*exo*,5-*endo*,8,8,10-pentachlorobornane.

There are only few weak intermolecular C—H···Cl and Cl···Cl contacts found in the structure. The static disorder of the present structure may be a consequence of intermolecular repulsive interactions between chlorines, found between two major components, but no unarguable evidence of this was found from the close contacts. However, the major component presented in Fig 1 has a close Cl···Cl contact of 3.49 Å between Cl2 and Cl5 of its enantiomer at -*x*, 2 - *y*, 1 - *z*. The same contanct is found also between Cl2 of the enantiomer and Cl5 in Fig 1, but between the minor components the contact (Cl2···Cl5b) is a bit longer (3.60 Å). The second difference found in the close contacts between major and minor components was another similar double interaction of major components between Cl5 and H10A at -*x*, 1 - *y*, 1 - *z*. This weak contact (2.59 Å) was not found between the minor components, in which the distance Cl5b···H10*c* is 3.60 Å.

### Experimental

The title compound was prepared *via* the following steps presented in Fig 2. First 6-*exo*-chlorocamphene was prepared according to a literature method (Jennings & Herschbach, 1965). Then crude 6-*exo*-chlorocamphene (5 g, 29 mmol) was dissolved in 50 ml of carbon tetrachloride. Chlorine gas was passed through this solution with stirring. After the solution obtained showed a persistent green colour, SnCl_4_ (1 g, 4 mmol) was added to the reaction mixture and passing of chlorine and stirring was continued for 4 h. The reaction mixture was washed with water and dried over calcium chloride. The solvent was removed in vacuo and the residue crystallized twice from hexane. The yield of the title compound was 2.2 g (25%, mp. 112 °C). ^1^H NMR (500 MHz, in CDCl_3_): 5.98 (H8), 4.43 (H5), 4.37 (H2), 3.89 (H10*a*), 3.81 (H10*b*), 3.02 (H3a), 2.63 (H6a), 2.55 (H4), 2.27 (H3b), 1.91 (H6b), 1.62 (3*H*, H9). ^13^C NMR (126 MHz, in CDCl_3_): 76.3 (C8), 63.1 (C2), 61.9 (C1), 56.7 (C7), 55.0 (C5), 54.5 (C4), 44.4 (2 C, C6 & C10), 33.6 (C3), 12.2 (C9). The obtained crystals were suitable for single-crystal X-ray structure determination.

### Refinement

The title compound shows static disorder and it was necessary to divide almost half the molecule in two partitions (Fig 3) as well as to use a large number of restraints (see below). The low data quality seems to be, however, not directly linked to the disorder. Refinement with or without the disorder taken into account (the latter with unacceptably asymmetric thermal ellipsoids) did result in nearly the same refined *R* values. We thus decided to test for various types of twinning, but the crystals did not appear to be twinned. *DIRAX* (Duisenberg, 1992) found the correct cell with less than one hundred fitting reflections. With relaxed conditions more than three hundred reflections were fitting the lattice. The same cell was also found using reflections that did not fit the first unit cell found using the strict values, but these lattices were rotated with respect to the first by less than 2 ° and attempts to refine the structure as non-merohedrally twinned using the hklf 5 routine failed. Visually the crystals seem to be of good quality with no evident fragmentation, but some fragmentation can be seen when cutting the large crystals The multiple unit cells found have thus been attributed to fragmentation of the single-crystal rather than non merohedral twinning. Several data collection endeavours with different crystals resulted in very similar results.

Very large and asymmetric thermal ellipsoids for several atoms indicated static disorder and atoms Cl1, Cl5, C1, C2, C3 and C10 and hydrogen atoms bonded to C2, C3, C4, C6 and C10 were refined as disordered over two partially occupied positions (Fig 3), with an occupancy ratio of 0.575 (major) to 0.425 (minor). The interatomic distances of non-hydrogen atoms of both partitions were restrained to be similar (SADI restraints with default standard deviations). The ADPs of atoms C1, C2, C3, C4, C6, C10, Cl1 and Cl2 (major) and the corresponding atoms of the minor component were restrained to be similar to those of their neighbors (SIMU and DELU restraints with default standard deviations). The ADPs of the disordered atoms were also restrained to be close to isotropic (ISOR restraints with s equal to 0.01 for C1, C2, C3, C10 (major), C1b, C2b, C3b and C10*b* (minor) and equal to 0.1 for Cl1, Cl5, Cl1b and Cl5b). The anisotropic displacement parameters of C1 and C1b set to be identical.

All H atoms were visible in electron density maps, but were placed in idealized positions and allowed to ride on their parent atoms at C—H distances of 0.98 (methyl), 0.99 (methylene) and 1.00 Å (methine), with *U*_iso_(H) of 1.5 (methyl) and 1.2 times *U*_eq_(C).

### Crystal data

**Table:**

C_10_H_13_Cl_5_	*D*_x_ = 1.610 Mg m^−^^3^
*M**_r_* = 310.45	Melting point: 385 K
Orthorhombic, *P**b**c**a*	Mo *K*α radiation λ = 0.71073 Å
Hall symbol: -P 2ac 2ab	Cell parameters from 61337 reflections
*a* = 12.2386 (2) Å	θ = 0.4–28.3º
*b* = 9.07010 (10) Å	µ = 1.10 mm^−^^1^
*c* = 23.0822 (3) Å	*T* = 173 (2) K
*V* = 2562.25 (6) Å^3^	Block, colourless
*Z* = 8	0.24 × 0.16 × 0.10 mm
*F*_000_ = 1264	

### Data collection

**Table:**

Bruker Kappa APEXII diffractometer	2612 independent reflections
Radiation source: fine-focus sealed tube	2440 reflections with *I* > 2σ(*I*)
Monochromator: graphite	*R*_int_ = 0.064
Detector resolution: 9 pixels mm^-1^	θ_max_ = 26.4º
*T* = 173(2) K	θ_min_ = 2.4º
φ and ω scans	*h* = −15→15
Absorption correction: multi-scan(MULABS in PLATON; Blessing, 1995; Spek, 2003)	*k* = −10→11
*T*_min_ = 0.779, *T*_max_ = 0.898	*l* = −28→27
35431 measured reflections	

### Refinement

**Table:**

Refinement on *F*^2^	Secondary atom site location: difference Fourier map
Least-squares matrix: full	Hydrogen site location: inferred from neighbouring sites
*R*[*F*^2^ > 2σ(*F*^2^)] = 0.091	H-atom parameters constrained
*wR*(*F*^2^) = 0.191	*w* = 1/[σ^2^(*F*_o_^2^) + 27.3151*P*] where *P* = (*F*_o_^2^ + 2*F*_c_^2^)/3
*S* = 1.26	(Δ/σ)_max_ < 0.001
2612 reflections	Δρ_max_ = 0.88 e Å^−^^3^
185 parameters	Δρ_min_ = −0.80 e Å^−^^3^
167 restraints	Extinction correction: none
Primary atom site location: structure-invariant direct methods	

### Special details

**Table:**

Geometry. All e.s.d.'s (except the e.s.d. in the dihedral angle between two l.s. planes) are estimated using the full covariance matrix. The cell e.s.d.'s are taken into account individually in the estimation of e.s.d.'s in distances, angles and torsion angles; correlations between e.s.d.'s in cell parameters are only used when they are defined by crystal symmetry. An approximate (isotropic) treatment of cell e.s.d.'s is used for estimating e.s.d.'s involving l.s. planes.
Refinement. Refinement of *F*^2^ against ALL reflections. The weighted *R*-factor *wR* and goodness of fit *S* are based on *F*^2^, conventional *R*-factors *R* are based on *F*, with *F* set to zero for negative *F*^2^. The threshold expression of *F*^2^ > σ(*F*^2^) is used only for calculating *R*-factors(gt) *etc*. and is not relevant to the choice of reflections for refinement. *R*-factors based on *F*^2^ are statistically about twice as large as those based on *F*, and *R*- factors based on ALL data will be even larger.

### Fractional atomic coordinates and isotropic or equivalent isotropic displacement parameters (Å^2^)

**Table:**

	*x*	*y*	*z*	*U*_iso_*/*U*_eq_	Occ. (<1)
C1	0.0424 (15)	0.7026 (18)	0.5949 (9)	0.0283 (17)	0.58 (5)
C2	0.1274 (12)	0.8287 (19)	0.5994 (11)	0.033 (3)	0.58 (5)
H2	0.1367	0.8786	0.5611	0.040*	0.58 (5)
C3	0.0694 (11)	0.933 (2)	0.6431 (10)	0.036 (4)	0.58 (5)
H3A	0.1136	0.9426	0.6789	0.043*	0.58 (5)
H3B	0.0605	1.0324	0.6259	0.043*	0.58 (5)
C10	0.0790 (16)	0.5631 (17)	0.5651 (9)	0.030 (4)	0.58 (5)
H10A	0.0142	0.5023	0.5558	0.036*	0.58 (5)
H10B	0.1258	0.5058	0.5919	0.036*	0.58 (5)
Cl1	0.2573 (10)	0.7613 (16)	0.6257 (11)	0.073 (4)	0.58 (5)
Cl5	0.1540 (13)	0.6004 (11)	0.4994 (7)	0.064 (3)	0.58 (5)
C1B	0.046 (2)	0.722 (3)	0.5991 (12)	0.0283 (17)	0.42 (5)
C2B	0.1255 (15)	0.848 (3)	0.6172 (13)	0.032 (4)	0.42 (5)
H2B	0.1401	0.9060	0.5812	0.038*	0.42 (5)
C3B	0.0629 (16)	0.951 (3)	0.6584 (13)	0.036 (5)	0.42 (5)
H3B1	0.0960	0.9553	0.6976	0.044*	0.42 (5)
H3B2	0.0552	1.0522	0.6426	0.044*	0.42 (5)
C10B	0.103 (2)	0.580 (3)	0.5818 (12)	0.033 (5)	0.42 (5)
H10C	0.0492	0.5000	0.5770	0.040*	0.42 (5)
H10D	0.1561	0.5509	0.6122	0.040*	0.42 (5)
Cl1B	0.2570 (13)	0.795 (3)	0.6450 (13)	0.072 (5)	0.42 (5)
Cl5B	0.174 (2)	0.613 (2)	0.5141 (13)	0.082 (6)	0.42 (5)
Cl2	−0.12193 (14)	1.06930 (17)	0.57587 (8)	0.0370 (4)	
Cl3	−0.08571 (17)	0.4090 (2)	0.65640 (9)	0.0484 (5)	
Cl4	−0.20761 (15)	0.6378 (3)	0.71306 (9)	0.0519 (6)	
C4	−0.0439 (6)	0.8662 (7)	0.6575 (3)	0.0340 (14)	
H4B	−0.0899	0.8915	0.6920	0.041*	0.42 (5)
H4A	−0.0797	0.9043	0.6935	0.041*	0.58 (5)
C5	−0.1096 (5)	0.8816 (6)	0.6013 (3)	0.0261 (13)	
H5	−0.1842	0.8390	0.6071	0.031*	
C6	−0.0450 (5)	0.7843 (7)	0.5581 (3)	0.0308 (13)	
H6C	−0.0136	0.8434	0.5261	0.037*	0.42 (5)
H6D	−0.0911	0.7046	0.5419	0.037*	0.42 (5)
H6A	−0.0098	0.8459	0.5281	0.037*	0.58 (5)
H6B	−0.0944	0.7130	0.5389	0.037*	0.58 (5)
C7	−0.0150 (5)	0.6992 (7)	0.6571 (3)	0.0264 (13)	
C8	−0.1180 (5)	0.6021 (7)	0.6551 (3)	0.0295 (14)	
H8	−0.1576	0.6236	0.6181	0.035*	
C9	0.0549 (6)	0.6536 (9)	0.7084 (3)	0.0469 (19)	
H9A	0.1201	0.7162	0.7102	0.070*	
H9B	0.0770	0.5504	0.7040	0.070*	
H9C	0.0127	0.6649	0.7443	0.070*	

### Atomic displacement parameters (Å^2^)

**Table:**

	*U*^11^	*U*^22^	*U*^33^	*U*^12^	*U*^13^	*U*^23^
C1	0.027 (3)	0.019 (4)	0.039 (4)	0.001 (3)	0.009 (2)	0.002 (3)
C2	0.028 (4)	0.028 (5)	0.044 (7)	−0.002 (4)	0.000 (5)	0.002 (5)
C3	0.043 (5)	0.023 (6)	0.042 (8)	−0.010 (4)	−0.015 (5)	−0.006 (5)
C10	0.034 (6)	0.023 (5)	0.034 (7)	−0.001 (4)	0.008 (5)	0.003 (5)
Cl1	0.024 (2)	0.049 (4)	0.148 (11)	−0.008 (2)	−0.010 (4)	0.028 (5)
Cl5	0.097 (6)	0.031 (3)	0.063 (5)	0.004 (4)	0.054 (5)	0.002 (3)
C1B	0.027 (3)	0.019 (4)	0.039 (4)	0.001 (3)	0.009 (2)	0.002 (3)
C2B	0.027 (5)	0.032 (7)	0.037 (8)	−0.005 (5)	0.001 (5)	0.010 (6)
C3B	0.044 (6)	0.028 (7)	0.037 (9)	−0.008 (5)	−0.011 (6)	−0.008 (6)
C10B	0.036 (8)	0.031 (6)	0.033 (8)	0.006 (6)	0.008 (6)	0.001 (6)
Cl1B	0.026 (3)	0.070 (10)	0.120 (12)	−0.009 (5)	−0.013 (5)	0.060 (9)
Cl5B	0.091 (9)	0.077 (9)	0.078 (10)	0.056 (7)	0.062 (8)	0.031 (7)
Cl2	0.0420 (9)	0.0262 (8)	0.0426 (9)	0.0103 (7)	0.0038 (7)	0.0046 (7)
Cl3	0.0581 (12)	0.0293 (9)	0.0577 (12)	−0.0116 (8)	0.0108 (9)	0.0093 (8)
Cl4	0.0378 (9)	0.0703 (13)	0.0475 (10)	0.0127 (9)	0.0193 (8)	0.0182 (10)
C4	0.038 (3)	0.031 (3)	0.033 (3)	0.004 (3)	−0.003 (3)	−0.003 (3)
C5	0.024 (3)	0.024 (3)	0.030 (3)	0.003 (2)	−0.001 (2)	0.002 (2)
C6	0.039 (3)	0.028 (3)	0.025 (3)	0.002 (3)	0.003 (3)	0.001 (3)
C7	0.028 (3)	0.022 (3)	0.030 (3)	0.000 (2)	−0.002 (3)	0.000 (2)
C8	0.022 (3)	0.033 (3)	0.034 (3)	−0.004 (3)	0.002 (3)	0.008 (3)
C9	0.041 (4)	0.045 (4)	0.055 (5)	−0.005 (3)	−0.022 (4)	0.013 (4)

### Geometric parameters (Å, °)

**Table:**

C1—C10	1.507 (13)	C10B—Cl5B	1.811 (14)
C1—C2	1.550 (14)	C10B—H10C	0.9900
C1—C6	1.553 (12)	C10B—H10D	0.9900
C1—C7	1.60 (3)	Cl2—C5	1.807 (6)
C2—C3	1.556 (16)	Cl3—C8	1.796 (7)
C2—Cl1	1.808 (11)	Cl4—C8	1.760 (7)
C2—H2	1.0000	C4—C5	1.533 (9)
C3—C4	1.550 (13)	C4—C7	1.556 (9)
C3—H3A	0.9900	C4—H4B	1.0000
C3—H3B	0.9900	C4—H4A	1.0000
C10—Cl5	1.806 (11)	C5—C6	1.549 (8)
C10—H10A	0.9900	C5—H5	1.0000
C10—H10B	0.9900	C6—H6C	0.9900
C1B—C10B	1.515 (15)	C6—H6D	0.9900
C1B—C7	1.55 (3)	C6—H6A	0.9900
C1B—C2B	1.557 (15)	C6—H6B	0.9900
C1B—C6	1.569 (15)	C7—C9	1.519 (9)
C2B—C3B	1.542 (18)	C7—C8	1.538 (8)
C2B—Cl1B	1.798 (14)	C8—H8	1.0000
C2B—H2B	1.0000	C9—H9A	0.9800
C3B—C4	1.519 (15)	C9—H9B	0.9800
C3B—H3B1	0.9900	C9—H9C	0.9800
C3B—H3B2	0.9900		
			
C10—C1—C2	116.8 (15)	C3B—C4—H4B	110.9
C10—C1—C6	110.9 (13)	C5—C4—H4B	110.9
C2—C1—C6	98.5 (11)	C3—C4—H4B	125.7
C10—C1—C7	121.5 (14)	C7—C4—H4B	110.9
C2—C1—C7	104.4 (12)	C3B—C4—H4A	100.9
C6—C1—C7	101.3 (12)	C5—C4—H4A	116.2
C1—C2—C3	100.8 (11)	C3—C4—H4A	115.7
C1—C2—Cl1	111.3 (11)	C7—C4—H4A	116.2
C3—C2—Cl1	112.9 (12)	C4—C5—C6	103.0 (5)
C1—C2—H2	110.5	C4—C5—Cl2	113.9 (4)
C3—C2—H2	110.5	C6—C5—Cl2	111.7 (4)
Cl1—C2—H2	110.5	C4—C5—H5	109.3
C4—C3—C2	108.0 (11)	C6—C5—H5	109.3
C4—C3—H3A	110.1	Cl2—C5—H5	109.3
C2—C3—H3A	110.1	C5—C6—C1	105.8 (9)
C4—C3—H3B	110.1	C5—C6—C1B	100.4 (12)
C2—C3—H3B	110.1	C5—C6—H6C	111.7
H3A—C3—H3B	108.4	C1—C6—H6C	113.4
C1—C10—Cl5	112.1 (13)	C1B—C6—H6C	111.7
C1—C10—H10A	109.2	C5—C6—H6D	111.7
Cl5—C10—H10A	109.2	C1—C6—H6D	104.6
C1—C10—H10B	109.2	C1B—C6—H6D	111.7
Cl5—C10—H10B	109.2	H6C—C6—H6D	109.5
H10A—C10—H10B	107.9	C5—C6—H6A	110.6
C10B—C1B—C7	109.9 (18)	C1—C6—H6A	110.6
C10B—C1B—C2B	113.7 (18)	C1B—C6—H6A	108.5
C7—C1B—C2B	99.7 (16)	H6D—C6—H6A	113.2
C10B—C1B—C6	118.4 (18)	C5—C6—H6B	110.4
C7—C1B—C6	103.1 (16)	C1—C6—H6B	110.7
C2B—C1B—C6	109.9 (15)	C1B—C6—H6B	117.9
C3B—C2B—C1B	107.6 (15)	H6C—C6—H6B	104.9
C3B—C2B—Cl1B	112.8 (16)	H6A—C6—H6B	108.7
C1B—C2B—Cl1B	117.3 (15)	C9—C7—C8	109.2 (5)
C3B—C2B—H2B	106.1	C9—C7—C1B	116.0 (7)
C1B—C2B—H2B	106.1	C8—C7—C1B	116.5 (10)
Cl1B—C2B—H2B	106.1	C9—C7—C4	112.8 (6)
C4—C3B—C2B	96.3 (14)	C8—C7—C4	111.8 (5)
C4—C3B—H3B1	112.5	C1B—C7—C4	89.2 (9)
C2B—C3B—H3B1	112.5	C9—C7—C1	117.3 (7)
C4—C3B—H3B2	112.5	C8—C7—C1	110.1 (8)
C2B—C3B—H3B2	112.5	C4—C7—C1	95.0 (7)
H3B1—C3B—H3B2	110.0	C7—C8—Cl4	112.5 (5)
C1B—C10B—Cl5B	108.0 (19)	C7—C8—Cl3	112.2 (4)
C1B—C10B—H10C	110.1	Cl4—C8—Cl3	107.7 (3)
Cl5B—C10B—H10C	110.1	C7—C8—H8	108.1
C1B—C10B—H10D	110.1	Cl4—C8—H8	108.1
Cl5B—C10B—H10D	110.1	Cl3—C8—H8	108.1
H10C—C10B—H10D	108.4	C7—C9—H9A	109.5
C3B—C4—C5	114.6 (13)	C7—C9—H9B	109.5
C5—C4—C3	104.6 (10)	H9A—C9—H9B	109.5
C3B—C4—C7	107.4 (12)	C7—C9—H9C	109.5
C5—C4—C7	101.7 (5)	H9A—C9—H9C	109.5
C3—C4—C7	100.2 (8)	H9B—C9—H9C	109.5
			
C10—C1—C2—C3	−168.0 (16)	C10B—C1B—C6—C1	21 (15)
C6—C1—C2—C3	73.3 (15)	C7—C1B—C6—C1	−100 (17)
C7—C1—C2—C3	−30.7 (13)	C2B—C1B—C6—C1	154 (18)
C10—C1—C2—Cl1	−48 (2)	C10B—C1B—C7—C9	59.1 (17)
C6—C1—C2—Cl1	−166.6 (12)	C2B—C1B—C7—C9	−60.6 (12)
C7—C1—C2—Cl1	89.3 (11)	C6—C1B—C7—C9	−173.8 (9)
C1—C2—C3—C4	−3.0 (16)	C10B—C1B—C7—C8	−71.6 (16)
Cl1—C2—C3—C4	−121.8 (13)	C2B—C1B—C7—C8	168.6 (9)
C2—C1—C10—Cl5	−44 (2)	C6—C1B—C7—C8	55.4 (15)
C6—C1—C10—Cl5	67.4 (18)	C10B—C1B—C7—C4	174.3 (15)
C7—C1—C10—Cl5	−173.8 (11)	C2B—C1B—C7—C4	54.5 (10)
C10B—C1B—C2B—C3B	−154 (2)	C6—C1B—C7—C4	−58.7 (12)
C7—C1B—C2B—C3B	−37.6 (18)	C10B—C1B—C7—C1	−43 (6)
C6—C1B—C2B—C3B	70 (2)	C2B—C1B—C7—C1	−162 (7)
C10B—C1B—C2B—Cl1B	−26 (3)	C6—C1B—C7—C1	84 (6)
C7—C1B—C2B—Cl1B	90.8 (16)	C3B—C4—C7—C9	57.4 (14)
C6—C1B—C2B—Cl1B	−161.4 (17)	C5—C4—C7—C9	178.1 (6)
C1B—C2B—C3B—C4	0(2)	C3—C4—C7—C9	70.8 (11)
Cl1B—C2B—C3B—C4	−130.7 (17)	C3B—C4—C7—C8	−179.0 (13)
C7—C1B—C10B—Cl5B	−179.7 (13)	C5—C4—C7—C8	−58.3 (7)
C2B—C1B—C10B—Cl5B	−69 (3)	C3—C4—C7—C8	−165.7 (10)
C6—C1B—C10B—Cl5B	62 (3)	C3B—C4—C7—C1B	−60.6 (13)
C2B—C3B—C4—C5	−73.8 (18)	C5—C4—C7—C1B	60.1 (8)
C2B—C3B—C4—C3	−24 (6)	C3—C4—C7—C1B	−47.3 (11)
C2B—C3B—C4—C7	38.4 (18)	C3B—C4—C7—C1	−65.0 (13)
C2—C3—C4—C3B	157 (8)	C5—C4—C7—C1	55.8 (7)
C2—C3—C4—C5	−68.9 (14)	C3—C4—C7—C1	−51.6 (10)
C2—C3—C4—C7	36.2 (14)	C10—C1—C7—C9	68.3 (15)
C3B—C4—C5—C6	74.6 (13)	C2—C1—C7—C9	−66.4 (10)
C3—C4—C5—C6	63.0 (9)	C6—C1—C7—C9	−168.4 (7)
C7—C4—C5—C6	−40.9 (6)	C10—C1—C7—C8	−57.4 (14)
C3B—C4—C5—Cl2	−46.6 (13)	C2—C1—C7—C8	167.8 (8)
C3—C4—C5—Cl2	−58.2 (9)	C6—C1—C7—C8	65.9 (10)
C7—C4—C5—Cl2	−162.1 (4)	C10—C1—C7—C1B	150 (7)
C4—C5—C6—C1	8.1 (10)	C2—C1—C7—C1B	15 (6)
Cl2—C5—C6—C1	130.7 (9)	C6—C1—C7—C1B	−87 (6)
C4—C5—C6—C1B	2.7 (13)	C10—C1—C7—C4	−172.8 (13)
Cl2—C5—C6—C1B	125.4 (12)	C2—C1—C7—C4	52.4 (9)
C10—C1—C6—C5	156.8 (13)	C6—C1—C7—C4	−49.5 (9)
C2—C1—C6—C5	−80.2 (13)	C9—C7—C8—Cl4	68.6 (6)
C7—C1—C6—C5	26.4 (11)	C1B—C7—C8—Cl4	−157.5 (8)
C10—C1—C6—C1B	−159 (18)	C4—C7—C8—Cl4	−56.9 (6)
C2—C1—C6—C1B	−36 (16)	C1—C7—C8—Cl4	−161.2 (6)
C7—C1—C6—C1B	71 (16)	C9—C7—C8—Cl3	−53.0 (7)
C10B—C1B—C6—C5	158 (2)	C1B—C7—C8—Cl3	80.9 (9)
C7—C1B—C6—C5	36.6 (14)	C4—C7—C8—Cl3	−178.5 (4)
C2B—C1B—C6—C5	−69 (2)	C1—C7—C8—Cl3	77.2 (7)
